# A novel proteomics workflow for simultaneous analysis of protein phosphorylation and *S*-nitrosylation

**DOI:** 10.1007/s42994-025-00227-2

**Published:** 2025-07-15

**Authors:** Wenyang Zhang, Yanjiao Wang, Wenyan Li, Shaowen Wu, Yuanyuan Chen, Mingyang Ye, Wenjie Huang, Alisdair R. Fernie, Shijuan Yan

**Affiliations:** 1 State Key Laboratory of Swine and Poultry Breeding Industry, Guangdong Key Laboratory for Crop Germplasm Resources Preservation and Utilization, Agro-Biological Gene Research Center, Guangdong Academy of Agricultural Sciences, Guangzhou, 510640 China; 2 State Key Laboratory for Conservation and Utilization of Subtropical Agro-Bioresources, Guangdong Laboratory for Lingnan Modern Agriculture, College of Life Sciences, South China Agricultural University, Guangzhou, 510642 China; 3 Max Planck Institute of Molecular Plant Physiology, Am Muhlenberg 1, 14476 Potsdam-Golm, Germany

**Keywords:** Proteomics, *S*-nitrosylation, Phosphorylation, PTM crosstalk

## Abstract

Protein post-translational modifications such as phosphorylation and *S*-nitrosylation regulate protein functions and cellular programs in eukaryotes. Moreover, extensive evidence suggests crosstalk between these modifications. However, we lack a comprehensive method for the simultaneous detection and analysis of multiple post-translational modifications. Here, we present an optimized workflow that identifies phosphorylation and *S*-nitrosylation sites using a novel phosphate affinity tag switch technique. Validation with model proteins and complex biological samples confirmed the high sensitivity, coverage, and reproducibility of this method. Applying this method to *Arabidopsis thaliana* seedlings revealed 12,552 phosphorylation sites and 6,108 *S*-nitrosylation sites, representing the largest single-study dataset of *S*-nitrosylation sites to date. This approach enhances our understanding of post-translational modification dynamics in plant signaling, stress responses, and metabolism.

**Supplementary Information:**

The online version contains supplementary material available at 10.1007/s42994-025-00227-2.

## Introduction

Post-translational modifications (PTMs) dynamically regulate protein functions and cellular activity. Among the more than 400 types of PTMs currently documented (Ramazi and Zahiri 2021), phosphorylation has been extensively studied due to its roles in signal transduction, protein–protein interactions, enzymatic activity, and subcellular localization in eukaryotes (Nishi et al. 2014). Similarly, *S*-nitrosylation, a redox-based PTM that covalently adds a nitric oxide (NO) moiety to reactive cysteine residues, has garnered increasing attention as a regulatory switch analogous to phosphorylation (Astier et al. 2011). Recent studies have explored the functions of *S*-nitrosylated proteins, underscoring their ubiquitous involvement in plant development and stress responses (Feng et al. 2019; Liu et al. 2024).

Proteins are often modified by different types of PTMs; crosstalk between these PTMs introduces an additional layer regulating protein function and enhances the complexity of the proteome (Hess and Stamler 2012; Leutert et al. 2021). For example, consistent with accumulating evidence of crosstalk between phosphorylation and *S*-nitrosylation, phosphorylation of SALICYLIC ACID-BINDING PROTEIN 3 (SABP3) requires its prior *S*-nitrosylation, whereas the phosphorylation of BOTRYTIS-INDUCED KINASE 1 (BIK1) precedes its *S*-nitrosylation to regulate immune signaling in Arabidopsis (*Arabidopsis thaliana*) (Cui et al. 2024; Rui et al. 2024). Beyond individual proteins, PTM of specific kinases influences signaling pathways by altering the phosphorylation of kinase substrates (Leutert et al. 2021). Indeed, *S*-nitrosylation modulates the activity of kinases, including members of the Sucrose-non-fermenting 1-related protein kinase 2 (SnRK2) family (Wang et al. 2015), TARGET OF RAPAMYCIN (TOR) (Lando et al. 2024), the receptor kinase FERONIA (FER) (Huang et al. 2023), MITOGEN-ACTIVATED PROTEIN KINASEs (MAPKs) (Bellin et al. 2013; Lv et al. 2017; Wei et al. 2022), and THIOREDOXIN-INTERACTING RECEPTOR KINASE (TIRK) (Arnaiz et al. 2023), suggesting that plant cell signaling involves complex crosstalk between *S*-nitrosylation and phosphorylation.

Despite growing interest in PTM crosstalk, technical challenges hinder the simultaneous analysis of *S*-nitrosylation and phosphorylation. For example, because of their low abundance, PTM-marked proteins must be enriched before they can be effectively detected by mass spectrometry (MS) (Feng et al. 2019). Moreover, the distinct chemical properties of phosphorylated and *S*-nitrosylated proteins necessitate separate enrichment strategies: metal oxide/ion affinity chromatography for phosphorylated proteins (Qiu et al. 2020) and tag switch techniques for *S*-nitrosylation (Zhou et al. 2023). Existing methods typically require independent or serial enrichment for each modification, which is impractical for small samples and introduces challenges such as variability in enrichment efficiency (Adoni et al. 2022), higher experimental complexity and costs, lower throughput, and potential sample loss due to non-specific adsorption (Eyrich et al. 2011). Unified approaches have been developed for PTMs with similar properties, such as phosphorylation and glycosylation (Huang et al. 2021; Palmisano et al. 2012), but technical limitations have prevented the simultaneous analysis of phosphorylation and *S*-nitrosylation (Liang et al. 2024; Liu et al. 2024; Vu et al. 2018).

To overcome these limitations, we developed a concurrent analysis workflow in which we label *S*-nitrosylated cysteines with a phospho-affinity tag (PAT), thus allowing us to enrich *S*-nitrosylated peptides and phosphopeptides using immobilized metal ion affinity chromatography (IMAC), followed by co-analysis through liquid chromatography–tandem mass spectrometry (LC–MS/MS). An initial validation using model proteins demonstrated that this technique achieves sensitivity and coverage comparable with those of traditional enrichment methods for each modification. Applying this method to complex biological samples, we identified numerous previously unreported *S*-nitrosylation sites and uncovered potential PTM crosstalk in key regulatory proteins, highlighting the power of this approach for investigating plant signaling, stress responses, and metabolic regulation.

## Results

### Establishment of the PAT-switch method for dual PTM analysis

**Fig. 1:**
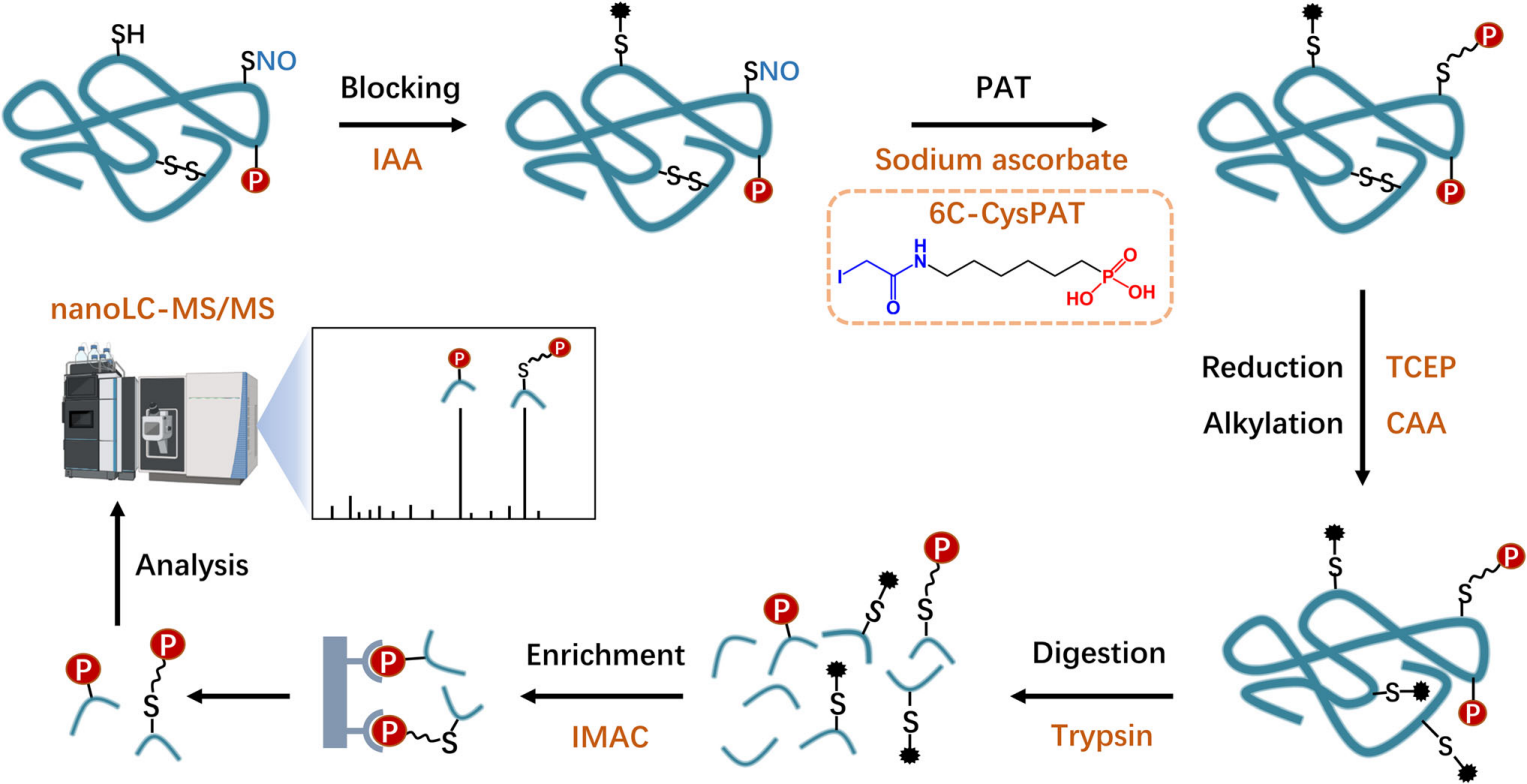
A stepwise illustration of the PAT-switch method for the simultaneous enrichment and identification of phosphorylated and *S*-nitrosylated proteins. The workflow includes: blocking free thiols, specific labeling of *S*-nitrosylated cysteines with the phosphate affinity tag (PAT), enzymatic digestion, IMAC enrichment of both modified peptides, and LC–MS/MS analysis

In this study, we developed a PAT-switch method, combining PAT labeling and the selective enrichment of phosphorylated and *S*-nitrosylated peptides (Fig. 1). This method consists of three strictly sequential steps: (1) blocking free thiols; (2) PAT labeling of *S*-nitrosylated thiols; and (3) reducing and alkylating disulfide bonds. We blocked free thiols using the classic S-alkylating reagent iodoacetamide (IAA), preserving *S*-nitrosylated cysteine and disulfide bonds. Excess iodoacetamide was removed through acetone precipitation, after which we selectively reduced *S*-nitrosylated cysteines with sodium ascorbate and labeled them with iodoacetamido-LC-phosphonic acid (6C-CysPAT). After methanol/chloroform precipitation to remove excess 6C-CysPAT, we reduced disulfide bonds and blocked them by adding 10 mM Tris(2-carboxyethyl)phosphine hydrochloride (TCEP) and 40 mM 2-chloroacetamide (CAA). Proteins were then enzymatically digested into peptides using trypsin, yielding a mixture of phosphopeptides, PAT-labeled peptides, and unmodified peptides. We used IMAC to simultaneously enrich phosphopeptides and PAT-labeled peptides, allowing unmodified peptides to be washed away. The enriched peptides were analyzed by liquid chromatography–tandem mass spectrometry (LC–MS/MS), with the distinct mass shifts (79.97 Da for phosphorylation, 221.08 Da for the PAT tag) enabling clear differentiation between the two PTMs.

### Validation of enrichment efficiency using a model protein

The enrichment efficiency of the IMAC-based phos-trap column for phosphopeptides was validated previously (Zhang et al. 2022). To evaluate its selective enrichment of PAT-labeled peptides, we used artificially *S*-nitrosylated bovine serum albumin (SNO-BSA) as a model substrate. Following the protocol of Wang et al. (Wang et al. 2024), the BSA was *S*-nitrosylated using sodium nitrite as a nitric oxide (NO) donor after reduction. The resulting SNO-BSA sample was divided into three equal portions to evaluate the effects of different treatments: (1) PAT labeling only; (2) PAT labeling followed by enrichment; and (3) untreated control.

**Fig. 2:**
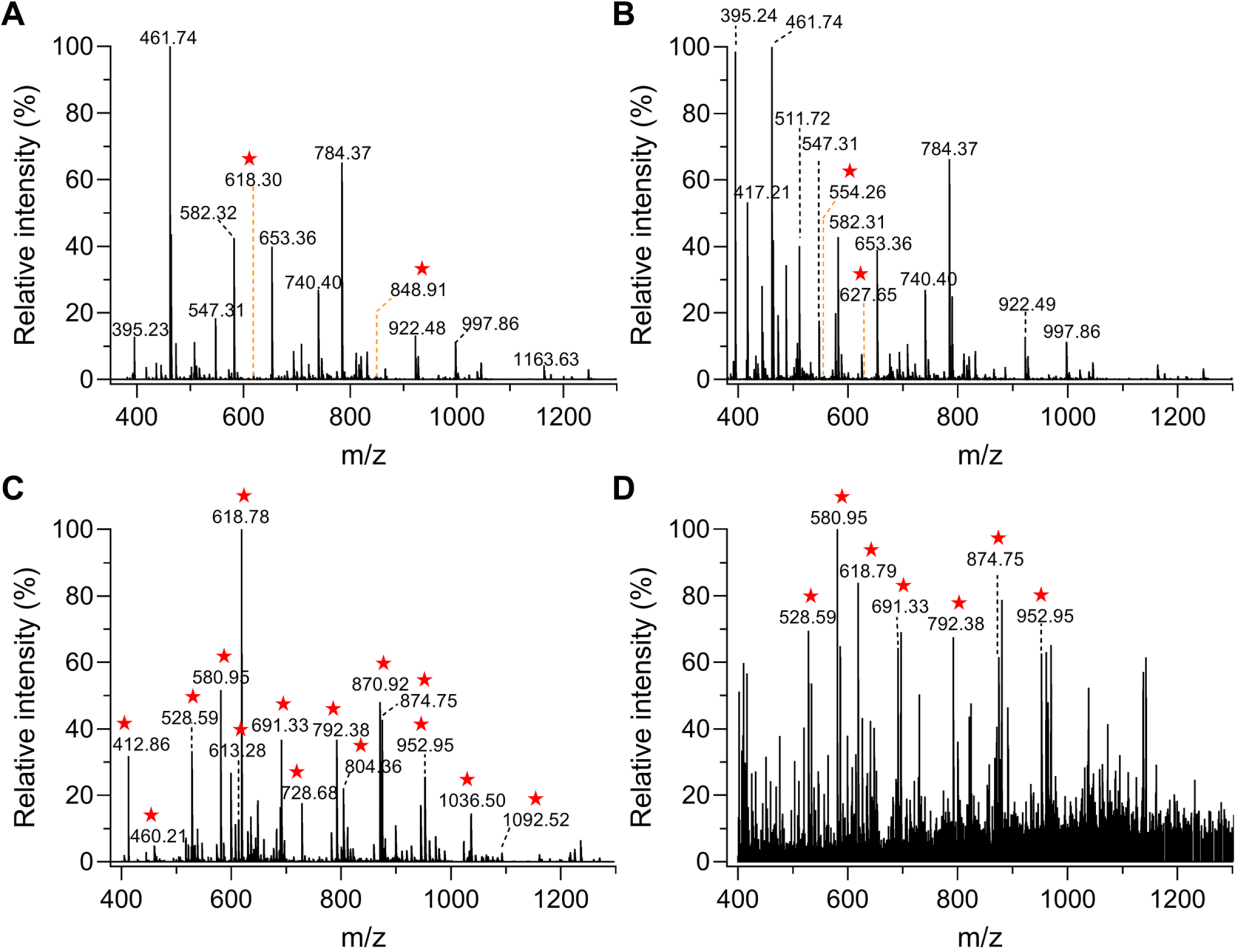
Selective enrichment of *S*-nitrosylated peptides from model protein SNO-BSA using the phos-trap column. **A–C** Mass spectra showing peptide compositions at different steps: **A** untreated, **B** PAT-labeled, and **C **PAT-labeled plus enrichment. **D** Sensitivity evaluation with 100 fmol SNO-BSA. Results demonstrate the high efficiency and specificity of the PAT-switch/IMAC method for S-nitrosylated peptide enrichment

Mass spectrometry analysis of the unenriched SNO-BSA digested samples revealed predominant signals from non-modified peptides and weak signals marked with red stars from *S*-nitrosylated peptides (Fig. 2A). PAT-based labeling did not substantially enhance the intensity of *S*-nitrosylated peptide signals (Fig. 2B). Notably, the PAT-labeled peptides demonstrated marked signal amplification only after enrichment via a phospho-trap column, with their signals now predominating in the mass spectrum (Fig. 2C; Supplementary Data 1). In total, we identified 30 *S*-nitrosylated sites among the 35 cysteine residues present in BSA. Despite their initial 1,000-fold greater abundance, the presence of non-modified peptides was effectively minimized after enrichment. A sensitivity analysis using trace SNO-BSA (100 fmol) detected seven PAT-labeled peptides (Fig. 2D), demonstrating the high specificity and sensitivity of our enrichment strategy.

### Assessment of method performance in complex biological samples

After validating the PAT and phos-trap enrichment method with SNO-BSA, we assessed its efficacy on biological samples for the simultaneous analysis of protein phosphorylation and *S*-nitrosylation, comparing the performance of our method against the cutting-edge *S*-nitrosoproteomics method fluorous affinity tag (FAT) (Qin et al. 2023) and the phosphoproteomics method GreenPhos (Duan et al. 2024). We processed protein samples extracted from Arabidopsis seedlings using the PAT-switch method, followed by single-injections in triplicate for online enrichment and LC–MS/MS analysis in data-dependent acquisition (DDA) mode.

**Fig. 3:**
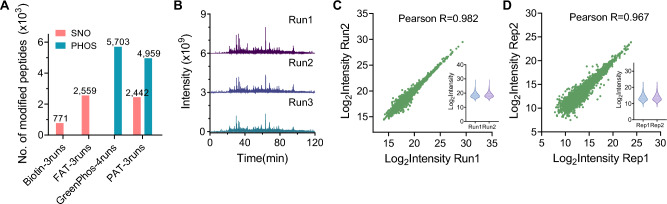
Evaluation of the PAT-switch workflow in *Arabidopsis* samples. **A** Comparison of peptide coverage for *S*-nitrosylation and phosphorylation against established single-omics workflows. **B** Total ion chromatograms (TICs) from triplicate runs illustrating analytical reproducibility. **C, D** Scatterplots of peptide abundances between (**C**) technical and (**D**) biological replicates, indicating high method reproducibility

When using 300 μg of protein extracted from Arabidopsis seedlings as the input sample, this technique identified 2,442 *S*-nitrosylated peptides and 4,959 phosphopeptides (Fig. 3A). The phosphopeptide coverage was slightly lower than that published for GreenPhos, which identified 5,703 phosphopeptides with an equivalent 300 μg protein input (Duan et al. 2024). The performance of the PAT-switch method in detecting *S*-nitrosylation was on par with that of FAT, which identified 2,559 *S*-nitrosylated peptides using 3 mg of protein in their work (Qin et al. 2023).

To further assess the applicability of our method when using low amounts of a sample (150 μg of protein extracted from 30 mg Arabidopsis seedlings), as well as the potential influence of PAT treatment and instrumental bias on phosphopeptide detection, we conducted a comparative analysis of PAT-treated and non-treated samples, with technical duplicates, following identical enrichment procedures and analysis by LC–MS/MS over a 120-min gradient separation. After data acquisition, we performed label-free quantification (LFQ) using the MS1 spectra with Proteome Discoverer™ 3.1, using the Minora Feature Detector node for peak detection, alignment, and cross-run matching. We detected 4,458 quantifiable phosphopeptides in the PAT-treated samples, against 4423 in non-treated controls, with 4005 phosphopeptides (or 82.1% of total) being consistently quantified across all samples (Fig. S1A, B). Notably, we identified 1693 *S*-nitrosylated peptides specifically in the PAT-treated samples (Fig. S1A). These results demonstrate the potential of our method for use with limited sample input and confirm that PAT treatment is compatible with phosphopeptide detection. In addition, we conclude that the additional enrichment and analysis of *S*-nitrosylated peptides do not exceed the capacity of either the affinity column or the mass spectrometer.

We evaluated the quantitative reproducibility of our method by running the same sample three times, yielding similar base peak chromatograms across the three runs (Fig. 3B). Following parallel enrichment and LC–MS/MS analysis, the log_2_-transformed intensities of modified peptides showed a high Pearson correlation coefficient between replicates, reaching 0.982 (Fig. 3C). Biological replicates also showed good reproducibility, with a Pearson correlation coefficient of 0.967 between them (Fig. 3D). The slight enhancement in the current method may originate from our previously developed online and automated phosphopeptide enrichment technology, which achieves precise control of reagent volume and flow rate, thereby improving the precision of parallel analyses (Zhang et al. 2022).

### Large-scale identification of phosphorylated and *S*-nitrosylated sites in Arabidopsis

To comprehensively identify *S*-nitrosylation (SNO) and phosphorylation sites in Arabidopsis samples, we employed a fractionation strategy utilizing high-pH reversed-phase high-pressure liquid chromatography (high-pH RP-HPLC) on the pretreated peptide mixtures to reduce sample complexity, after which consecutive fractions were pooled into 12 pools for separate analysis. We merged the resulting data from each pool and analyzed the merged data using Proteome Discoverer™ 3.1. The probability of each *S*-nitrosylation or phosphorylation site was assessed using ptmRS (Taus et al. 2011), with sites scoring above the 0.75 cutoff classified as Class I sites.

**Fig. 4:**
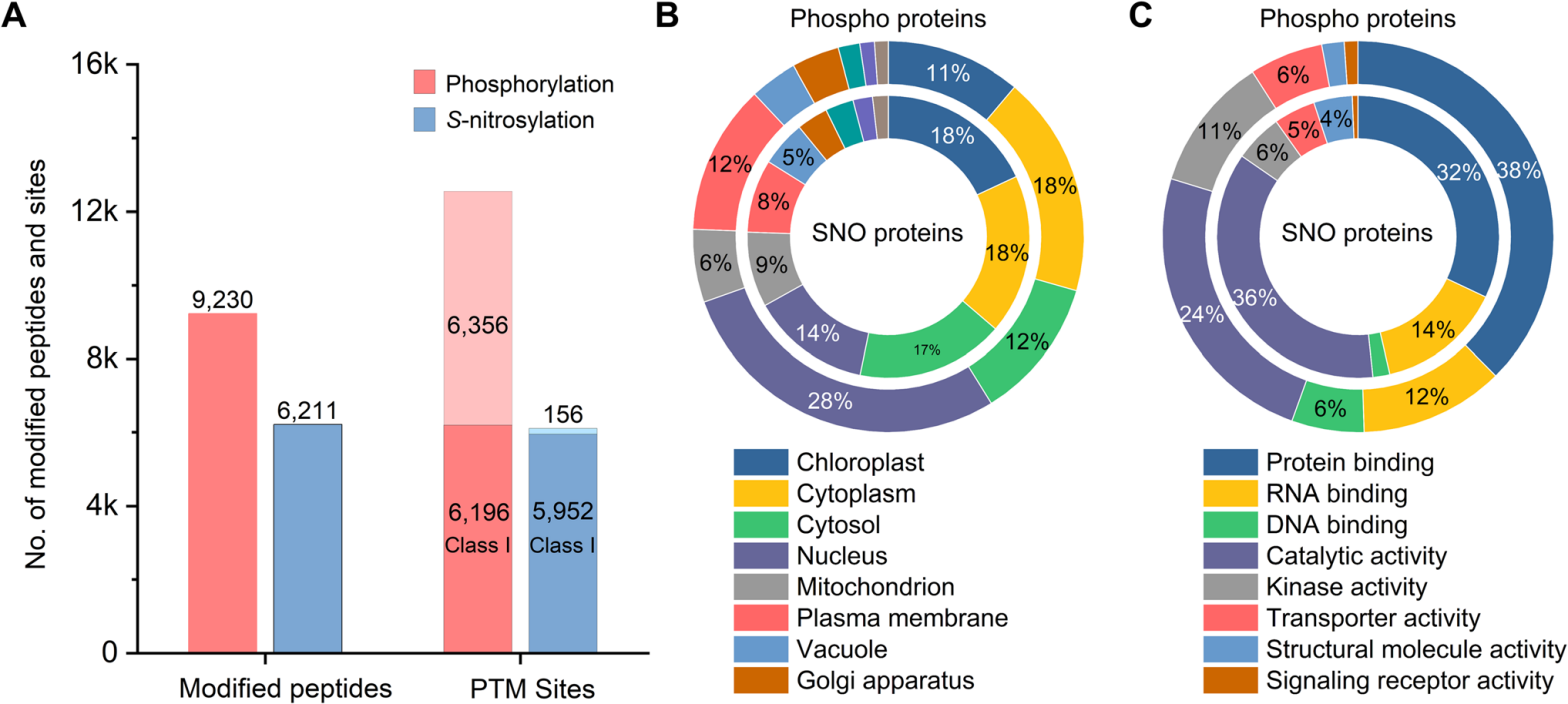
**A** Statistical analysis of *Arabidopsis* phosphorylation and *S*-nitrosylation identifications. **B** Subcellular localization of modified proteins. **C** Functional classification of modified proteins

Our global analysis revealed an extensive PTM landscape in Arabidopsis seedlings (Fig. 4A; Supplementary Data 2), identifying 12,552 phosphorylation sites distributed across 9,230 phosphopeptides, of which 6,196 were Class I sites. In addition, we detected 6,108 *S*-nitrosylation sites within 6,211 SNO peptides, with 5,952 classified as Class I sites.

To assess the novelty of our findings, we compared our data against established databases. Accordingly, cross-referencing our phosphorylation dataset with 187,832 previously documented phosphosites from three major databases, EPSD (Lin et al. 2020), qPTMplants (Xue et al. 2021), and Plant PTM Viewer 2.0 (Willems et al. 2024), revealed 618 Class I phosphosites currently not listed in these databases (Supplementary Data 3). Furthermore, we discovered 3795 previously unreported Class I *S*-nitrosylation sites by comparing our dataset with 9,044 known sites compiled from Plant PTM Viewer, qPTMplants, and iCysMod (Wang et al. 2021) (Supplementary Data 4).

The *S*-nitrosylated and phosphorylated proteins we identified here also showed distinct predicted subcellular locations and functions (Fig. 4B, C). *S*-nitrosylated proteins were predominantly predicted to localize in metabolically active organelles, with over 50% of the modified proteins identified collectively localizing to the chloroplast, cytoplasm, and cytosol. These proteins were strongly enriched for functions related to catalytic activity (36.3%) and protein/RNA binding (46.4% combined). By contrast, phosphorylated proteins were predicted to be more concentrated in the nucleus (27.3%) and at the plasma membrane (11.9%), with functions enriched in protein binding (37.6%) and kinase activity (11.1%). Notably, phosphorylated proteins showed a higher representation than *S*-nitrosylated proteins in DNA binding (6.0% vs. 1.9%) and kinase activity (11.1% vs. 5.5%).

### Identification of dual-modified proteins in Arabidopsis

We identified a total of 968 Arabidopsis proteins that are dual-modified by phosphorylation and *S-*nitrosylation (Supplementary Data 5). Among them, 378 proteins had not been previously identified as being dual-modified (labeled as “novel”), with previously unreported modification sites being identified in an additional 392 proteins (labeled as “new site found”) such as TOR, members of the phosphoenolpyruvate carboxylase family (PPC1, PPC2, PPC3), BRASSINOSTEROID-SIGNALING KINASE family members (BSK1, BSK3, BSK5–8), MAPK family members (MAPK2, MAPK6–9, MAPK15, MAPK16, MAPK18), and heat shock proteins (HSP70-1, HSP70-14, HSP70-15, HSP70-16).

**Fig. 5:**
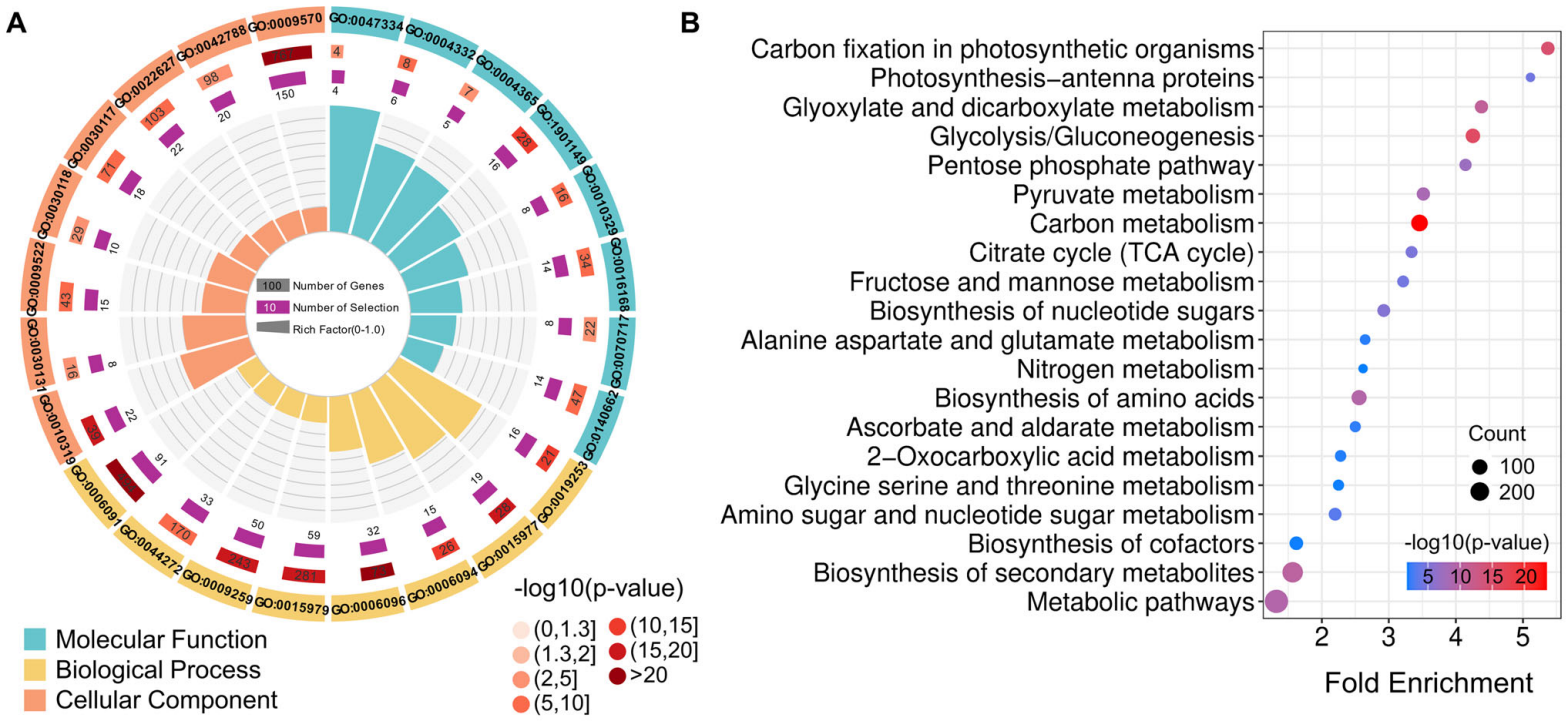
Functional enrichment analysis of dual-modified proteins in *Arabidopsis*. **A** Gene Ontology (GO) enrichment of proteins harboring both phosphorylation and *S*-nitrosylation, highlighting involvement in carbon fixation, glycolysis, and photosynthesis. **B** KEGG pathway analysis, indicating significant enrichment in metabolic and biosynthetic pathways

A gene ontology (GO) term enrichment analysis of the 968 dual-modified proteins revealed a strong association with photosynthesis, carbon metabolism, and other central metabolic pathways (Fig. 5A, with detailed names in Fig. S2). The most enriched biological processes included ‘carbon fixation’ (GO:0019253), ‘glycolysis’ (GO:0006096), and ‘photosynthesis’ (GO:0015979). Among the predicted cellular components, 150 dual-modified proteins localized to the chloroplasts, where light-dependent metabolism and carbon fixation take place. A GO term enrichment analysis for molecular function highlighted the involvement of these dual-modified proteins in various enzymatic activities, particularly in carbon metabolism and energy conversion, such as ‘fructose-bisphosphate aldolase activity’ (GO:0004332) and ‘glyceraldehyde-3-phosphate dehydrogenase activity’ (GO:0004365). We also noticed enrichment in phytohormone-related functions, including ‘salicylic acid binding’ (GO:1,901,149) and ‘auxin transport’ (GO:0010329), suggesting potential roles for these proteins in phytohormone signaling pathways. These findings suggest that proteins carrying both types of PTMs are predominantly involved in energy metabolism, phytohormone signaling pathways, and photosynthesis. The significant enrichment of these proteins in chloroplast-related components supports the function of these proteins in photosynthesis. Furthermore, the enrichment of phytohormone-related functions indicates potential crosstalk between these PTMs and phytohormone signaling networks.

A Kyoto Encyclopedia of Genes and Genomes (KEGG) pathway analysis supported these findings, revealing significant enrichment in metabolic pathways such as ‘carbon fixation in photosynthetic organisms,’ ‘glycolysis/gluconeogenesis,’ and ‘the tricarboxylic acid cycle’ (Fig. 5B). These results underscore the potential pivotal role of phosphorylation and *S*-nitrosylation in coordinating carbon flux and energy production. Moreover, we observed an enrichment for pathways related to amino acid biosynthesis, nucleotide sugar metabolism, and secondary metabolite biosynthesis, suggesting that those proteins that are dual-modified by phosphorylation and *S-*nitrosylation may influence a broad range of anabolic and catabolic pathways, potentially affecting plant growth and stress responses.

Together, these GO and KEGG analyses suggest that *S*-nitrosylation and phosphorylation work in concert to regulate key metabolic and photosynthetic functions in Arabidopsis. The convergence of these modifications on proteins central to carbon metabolism, energy generation, and biosynthetic pathways points to a complex regulatory network that fine-tunes plant physiology.

### Dual-modified transcription factors and kinases cooperate to modulate cellular signaling pathways

**Fig. 6:**
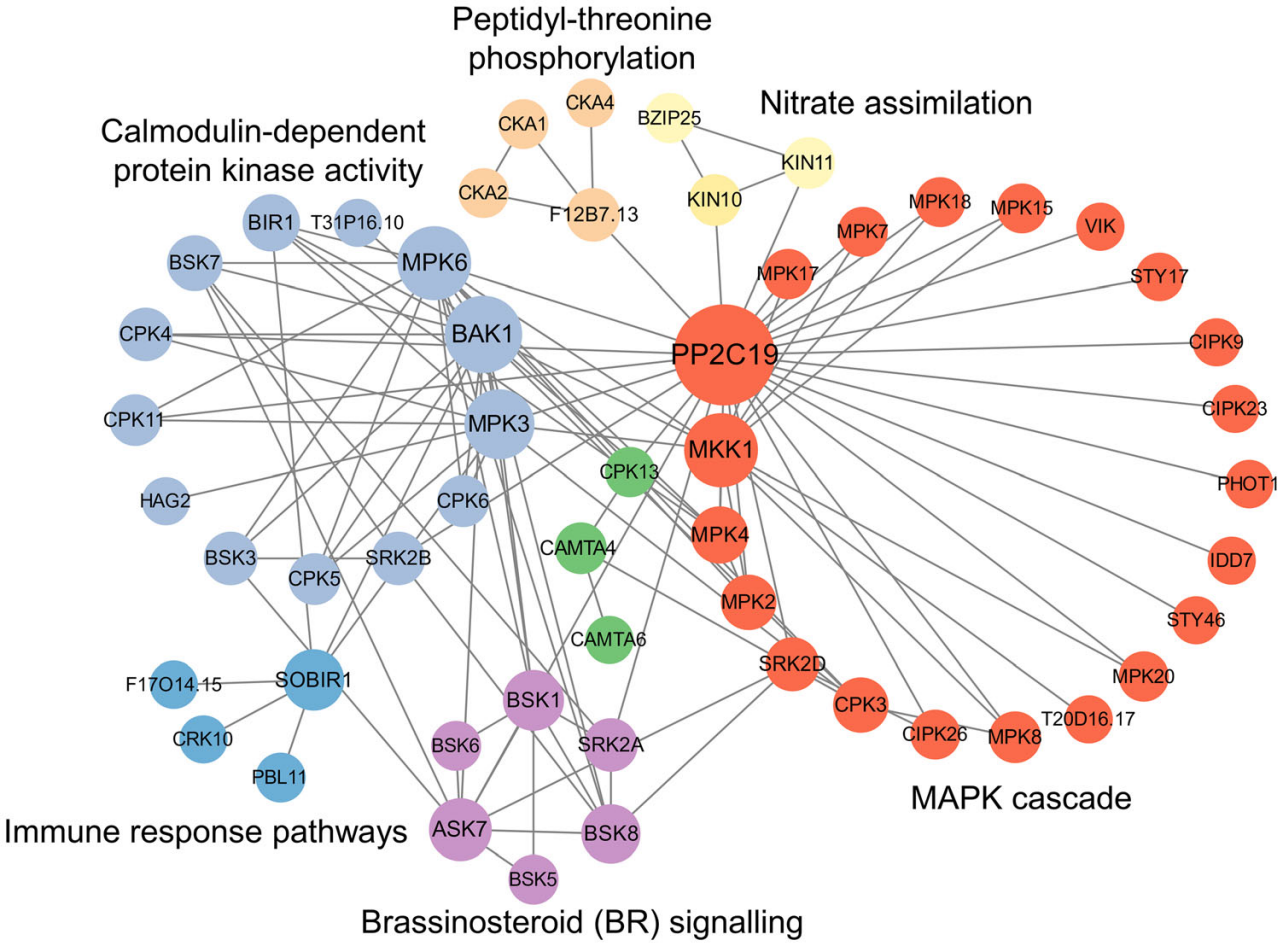
Protein–protein interaction (PPI) network of transcription factors, transcriptional regulators, and kinases with *S*-nitrosylation or dual-modifications. Network analysis reveals functionally interconnected modules among dual-modified proteins, suggesting coordinated regulation of signal transduction and stress response in *Arabidopsis*

In plants, transcription factors (TFs), transcriptional regulators (TRs), and protein kinases are key to the regulation of gene expression, signal transduction, and essential cellular programs (Zheng et al. 2016). We identified *S*-nitrosylation and phosphorylation sites for 17 TFs and 3 TRs (Table 1). Additionally, 74 kinases were *S*-nitrosylated, of which 56 were also phosphorylated, suggesting potential inter- and intra-protein crosstalk (Supplementary Data 6). A protein–protein interaction (PPI) network analysis of the 94 proteins (17 TFs + 3 TRs + 74 kinases; Fig. 6) revealed a highly interconnected network centered around the protein phosphatase PP2C19, which is potentially connected to six distinct functional modules: a MAPK cascade, brassinosteroid (BR) signaling, immune responses, calmodulin-dependent protein kinase activity, peptidyl-threonine phosphorylation, and nitrate assimilation. The MAPK cascade module showed extensive connections from PP2C19 to MPK7, MPK17, and MPK18, suggesting reciprocal regulation between phosphorylation and *S*-nitrosylation. Additionally, the BR signaling cluster (BSK1, ASK7, and BSK8) was linked to immune responses, which may support the idea that these two PTMs cooperate to modulate cellular responses. These findings offer new insights into the ways in which phosphorylation and *S*-nitrosylation coordinate protein function and cellular signaling.

**Table 1:** Summary of dual-modified transcription factors (TRs) and transcription regulators (TRs)

Accession	Protein name	Short name	Gene name	Category	SNO sites	PhosphoSites
AT1G35460.1	Basic helix-loop-helix protein 80	BH080	BHLH80	bHLH TF	C179	S75; S136
AT3G54620.2	Basic leucine zipper 25	bZIP25	BZIP25	bZIP TF	C294	S45; S71
AT1G55110.1	Protein indeterminate-domain 7	IDD7	IDD7	C2H2 TF	C97	S82
AT2G19810.1	Zinc finger CCCH domain-containing protein 20	C3H20	F6F22.16	C3H TF	C100	S197; S218; S233; S256; S275
AT2G40140.1	Zinc finger CCCH domain-containing protein 29	C3H29	T07M07.3	C3H TF	C236	S328; S438; S483; S485
AT2G41900.1	Zinc finger CCCH domain-containing protein 30	C3H30	T6D20.20	C3H TF	C283	S375; S496; S510; S541; S551; S566; S613
AT3G02830.1	Zinc finger CCCH domain-containing protein 33	C3H33	ZFN1	C3H TF	C91; C280; C341	S184; S219; Y251; S357
AT4G29190.1	Zinc finger CCCH domain-containing protein 49	C3H49	F17A13.10	C3H TF	C101	S187; S198; S271
AT5G58620.1	Zinc finger CCCH domain-containing protein 66	C3H66	MZN1.16	C3H TF	C231	S435
AT1G67310.1	Calmodulin-binding transcription activator 4	CMTA4	CAMTA4	CAMTA TF	C124	S773; S962
AT3G16940.1	Calmodulin-binding transcription activator 6	CMTA6	CAMTA6	CAMTA TF	C626	S158
AT2G21060.1	Cold shock domain-containing protein 4	CSP4	CSP4	CSD TF	C185	S169
AT2G35940.2	BEL1-like homeodomain protein 1	BLH1	BLH1	HB-BELL TF	C130	T132; S515
AT1G09770.1	Cell division cycle 5-like protein	Cdc5-like	CDC5	MYB TF	C107; C752	T226; T343; T399; T441
AT5G06110.1	Cell division related protein-like, DnaJ and myb-like DNA-binding domain-containing protein	/	ATGLSA2	MYB-related TF	C192	S530; S549
AT5G63420.1	Ribonuclease J (EC 3.1.-.-)	RNJ	RNJ	Trihelix TF	C125; C565; C631	S805
AT5G65410.1	Zinc-finger homeodomain protein 1	ZHD1	ZHD1	zf-HD TF	C229	S255
AT5G56740.1	Histone acetyltransferase type B catalytic subunit	HAT B	HAG2	GNAT TR	C295	S24
AT2G32700.7	LEUNIG_HOMOLOG	LUH	LUH	LUG TR	C729	S211
AT2G16485.1	Zinc finger CCCH domain-containing protein 19	C3H19	NERD	SWI/SNF-BAF60b TR	C1053	S1281; T1431

## Discussion

The identification of* S*-nitrosylated proteins traditionally relies on the biotin switch technique (Jaffrey and Snyder 2001). However, residual biotinylated reagents or streptavidin that co-elute from the affinity resin can interfere with MS/MS analysis. To address these limitations, other tag switch techniques have been developed, such as isotope coded affinity tag (ICAT) (Wu et al. 2011), bio-orthogonally cleavable Cys-BOOST (Mnatsakanyan et al. 2019), iodoacetyl tandem mass tag (iodo-TMT) (Gong and Shi 2019), and fluorous affinity tag (FAT) (Qin et al. 2023). However, these techniques are limited to *S*-nitrosylation, as phosphorylated peptides are excluded during selective enrichment. Here, we propose a strategy to convert *S*-nitrosylated sites into pseudo-phosphorylated sites using a phospho-affinity tag (PAT), enabling the enrichment and analysis of both PAT-labeled *S*-nitrosylated peptides and phosphopeptides using conventional phosphoproteomics techniques. The PAT reagent consists of three essential components: an iodoacetyl group with thiol reactivity, an alkyl spacer segment, and a specific phosphate tag. Originally designed for enriching Cys-containing peptides (Liu et al. 2023), we selected 6C-CysPAT as the reagent due to its commercial availability and validated reactivity toward thiol groups. Moreover, its application can be adapted for SNO-specific labeling using strategies such as Cys-BOOST or FAT, which involve free thiol blocking, SNO reduction and labeling, and disulfide bond reduction (Mnatsakanyan et al. 2019; Qin et al. 2023).

We defined the optimal conditions for CysPAT labeling as a reagent concentration of 5 mM and a reaction time of 60 min, as this combination yielded the highest number of identified *S*-nitrosylated peptides (Fig. S3) while conserving the use of the costly CysPAT reagent. To minimize the false discovery rate (FDR), we adopted the thiol-blocking reagent iodoacetamide, achieving over 97% site blocking efficiency when used at a concentration of 100 mM after 1 h of incubation (Fig. S4). Unblocked thiol sites, likely attributable to low site accessibility or interference, are unlikely to react with 6C-CysPAT at the low concentration employed (5 mM). A non-PAT sample yielded only five false positives, maintaining an FDR below 1‰ (Fig. S5).

One advantage of the method presented here is that it minimizes sample consumption. For instance, analysis of the two types of PTMs using GreenPhos (Duan et al. 2024) and FAT (Qin et al. 2023) typically requires 330 mg of plant tissue per sample. By contrast, our approach was successful using 30-mg samples. We attribute this sensitivity even with small sample sizes to the strong chelating effect of immobilized high-valent zirconium (Zr^4+^) ions toward phospho-affinity groups (Zhang et al. 2022), which also enables a highly efficient enrichment of PAT-labeled *S*-nitrosylated peptides, thereby substantially diminishing the required sample size. This improvement is particularly valuable in situations where sample availability is limited, particularly in studies involving rare species, specific tissues, developmental stages, or stress conditions.

Notably, our analysis indicates that the enrichment of PAT-labeled *S*-nitrosylated peptides in addition to phosphorylated peptides did not saturate the binding capacity of the phos-trap column, nor exceed the detection dynamic range of the mass spectrometer. This is consistent with prior studies demonstrating that multiplexed PTM detection strategies can be implemented without significant loss of targets (Basisty et al. 2018).

Limited coverage has posed a major challenge in *S*-nitrosoproteomics. Our method achieved coverage comparable to that of FAT (Qin et al. 2023) and GreenPhos (Duan et al. 2024), techniques developed for *S*-nitrosoproteomics and phosphoproteomics, respectively. This approach minimizes labor, reagent consumption, and instrument time compared with parallel or sequential single-omics analysis, and the consistency of both omics datasets provides more reliable biological insights.

In this study, we identified 12,552 phosphorylation sites (6205 Class I sites) and 6108 *S*-nitrosylation sites (5952 Class I sites). To our knowledge, this dataset represents the largest set of *S*-nitrosylation sites reported in a single proteomic study, with the discovery of 3795 *S*-nitrosylation sites not yet included in current databases, accounting for 42.0% of all currently known sites (9,044 known sites compiled from Plant PTM Viewer, qPTMplants, and iCysMod). Our findings indicate that *S*-nitrosylation is a global PTM comparable with phosphorylation, and undergoes considerable crosstalk.

Our comprehensive dual-modification analysis technique validated previously reported phosphorylation and *S*-nitrosylation in key proteins and signaling pathways. For instance, *S*-nitrosylation protects phosphoenolpyruvate carboxylase (PEPC), an enzyme integral to carbon and nitrogen metabolism, from inactivation under salinity stress while concurrently enhancing its phosphorylation (Baena et al. 2017). We identified dual modifications on multiple PEPC isoforms (PPC1, PPC2, and PPC3), with conserved sites, suggesting functional significance. Additionally, PTMs including phosphorylation, acetylation, and *S*-nitrosylation regulate molecular chaperones, such as HSP90 and HSP70 (Marozkina and Gaston 2012).

Our method uncovered previously unreported PTM patterns in signaling components, including a previously unreported *S*-nitrosylation site (Cys-49) on TOR (Lando et al. 2024), as well as on the BSK family members BSK1, BSK3, and BSK5–8. In the abscisic acid (ABA) signaling pathway, we detected both *S*-nitrosylation (Cys-160) and phosphorylation (Ser-30, Ser-152) on the kinase SNF1-related protein kinase 2.3 (SnRK2.3, also reported as SRK2I), as well as *S*-nitrosylation (Cys142) and phosphorylation (Ser-134, Ser-154) on SnRK2.10 (also known as SRK2B), which suggests potential regulatory crosstalk. This technique also facilitated the systematic identification of *S*-nitrosylation sites in MAPKs, including MAPK2, MAPK7, MAPK8, MAPK11, MAPK15–18, and MAPK20, potentially extending our understanding of dual PTM regulation in plant signaling. Taken together, these findings highlight the potential for simultaneously profiling two PTMs to uncover complex crosstalk in plant signaling pathways, providing deeper insights into their possible regulatory roles.

In this study, we developed a PAT-switch method to enable the simultaneous analysis of protein phosphorylation and *S*-nitrosylation sites. This method addresses the technical challenges of analyzing PTMs with distinct chemical properties, providing a reliable approach for comprehensive PTM crosstalk studies. Through extensive validation utilizing Arabidopsis samples, we demonstrated the high sensitivity, reproducibility, and broad coverage of this method across a wide range of proteins. The identification of 12,552 phosphorylation sites and 6,108 *S*-nitrosylation sites, including a substantial number that, to our knowledge, have not been previously reported, enhances the current understanding of the PTM landscape in plants. Moreover, the discovery of dual-modified proteins, particularly TFs and kinases, suggests potential mechanisms of PTM crosstalk in cellular signaling networks. Further research is warranted to elucidate the functional significance of these modifications, but our method provides a promising platform for future investigations of PTM-mediated regulation in plants and other biological systems.

## Materials and methods

### Reagents and materials

The following chemicals were purchased from Sigma-Aldrich (MO, USA): bovine serum albumin (BSA, Cat. No. V900933), iodoacetamide (Cat. No. V900335), 2-chloroacetamide (Cat. No. 22790), dithiothreitol (DTT, Cat. No. D0632), Tris (2-carboxyethyl)phosphine hydrochloride (TCEP, Cat. No. C4706), acetone (Cat. No. 650501), and chloroform (Cat. No. 319988). LC–MS grade acetonitrile (ACN, Cat. No. 047138-M1), water (Cat. No. 047146.K7), methanol (Cat. No. 019393-M1), formic acid (Cat. No. FA, 28,905), trifluoroacetic acid (Cat. No. 302031) iodoacetamide-LC-phosphonic acid (6C-CysPAT, Cat. No. A52285), protease and phosphatase inhibitor mini-tablets (Cat. No. A32961), a bicinchoninic acid (BCA) protein assay kit (Cat. No. 23227), Zeba protein desalting spin columns (Cat. No. 89890), Pierce peptide desalting spin columns (Cat. No. 89851), and Pierce Quantitative Colorimetric Peptide Assay (Cat. No. 23275) were obtained from Thermo Fisher Scientific (Waltham, MA, USA). HEPES (Cat. No. H433402), EDTA (Cat. No. E112488), neocuproine (Cat. No. N170560), sucrose (Cat. No. S112228), SDS (Cat. No. S108350), ammonium acetate (NH_4_OAc, Cat. No. A112057), sodium chloride (NaCl, Cat. No. C111547), sodium nitrite (NaNO_2_, Cat. No. S433708), hydrochloric acid (HCl, Cat. No. H466585), sodium ascorbate (Cat. No. S105024), urea (Cat. No. U141075), and ammonium bicarbonate (NH_4_HCO_2_, Cat. No. A431332) were purchased from Aladdin Scientific (Shanghai, China). Sequencing-grade trypsin was obtained from Promega Corporation (Madison, WI, USA, Cat. No. V5113).

The mixer mill (MM400) and sonicator (Bioruptor UCD-300) were purchased from RETSCH (Germany) and Diagenode (Belgium), respectively. The centrifuge (Multifuge 1S-R), nanoLC (Ultimate 3000 RSLCnano), and mass spectrometer (Orbitrap Fusion) were obtained from Thermo Fisher Scientific (Waltham, MA, USA).

### Preparation of artificially *S*-nitrosylated BSA

Artificially *S*-nitrosylated BSA (SNO-BSA) was prepared according to a recently published method (Wang et al. 2024), with modifications. Briefly, BSA was dissolved in 0.1 M Tris–HCl (pH 8.5) to a concentration of 5 mg·mL^−1^ and reduced through the addition of 20 mM Tris(2-carboxyethyl)phosphine hydrochloride at 37 °C for 30 min. After two rounds of desalting on Zeba spin columns, the solution was adjusted to pH 1.0 using HCl, and the solution was mixed with an equal volume of NaNO_2_ (at a ~ 20-fold molar excess relative to BSA thiols). The mixture was incubated in the dark at 25 °C for 1 h. The product was precipitated by the addition of four volumes of cold acetone and incubation at − 20 °C for 2 h. Proteins were pelleted through centrifugation at 8000 × *g* for 20 min at 4 °C; the pellet was washed successively with cold acetone and cold methanol.

### Whole protein extraction from Arabidopsis seedlings

All procedures were performed at low temperatures (on ice) under low-intensity light using metal-free reagents (unless otherwise specified). Arabidopsis seedlings were ground into a fine powder in liquid nitrogen using a mixer mill. About 0.2 g of powder was resuspended in 2 mL ice-cold HENS buffer (100 mM HEPES, pH 7.7, 1 mM EDTA, 0.1 mM neocuproine, 1% [w/v] SDS) containing 0.9 M sucrose, supplemented with protease/phosphatase inhibitors. Proteins were extracted through 10 cycles of sonication (10 s on/10 s off) in an ice bath. The homogenate was centrifuged at 16,000 × *g* for 15 min at 4 °C; the supernatant was transferred to a new tube. Iodoacetamide was added to a final concentration of 100 mM, followed by incubation at 37 °C for 1 h with shaking at 600 rpm. The solution was mixed with an equal volume of Tris-buffered phenol (pH 8.0), vortexed for 10 min, and centrifuged at 8000 × *g* for 20 min at 4 °C. The upper phenol phase was collected, mixed with five volumes of pre-cooled methanol containing 0.1 M NH_4_OAc, and incubated at − 20 °C for 3 h. Proteins were pelleted through centrifugation at 8000 × *g* for 20 min at 4 °C; the pellet was washed with cold methanol (containing 0.1 M NH_4_OAc) and 80% (v/v) acetone (containing 10 mM NaCl), before being air-dried for 5 min.

### PAT-switch labeling of SNO-proteins

All procedures were conducted under low-intensity light. The protein pellet was resuspended in 500 μL HENS buffer with 20 mM sodium ascorbate and 5 mM 6C-CysPAT. The mixture was incubated at 37 °C for 1 h with gentle rolling. Rapid protein precipitation was performed as described by Nickerson and Doucette (Nickerson and Doucette 2020). Briefly, the sample was vortexed with 50 μL of 1 M NaCl, followed by protein precipitation through the addition of 2 mL acetone and incubation at room temperature for 30 min. Following centrifugation at 8000 × *g* for 10 min at 4 °C, the protein pellet was washed twice with 80% (v/v) acetone containing 10 mM NaCl and air-dried for 5 min.

### Protein reduction, alkylation, and enzymatic digestion

As residual SDS would interfere with subsequent enrichment of phosphopeptides and PAT-labeled peptides, the use of SDS-containing buffers was avoided for the follow-up steps. Protein pellets were resuspended in 0.4 mL denaturing buffer (8 M urea, 50 mM ammonium bicarbonate, 10 mM Tris(2-carboxyethyl)phosphine hydrochloride, and 40 mM 2-chloroacetamide) and incubated at 60 °C for 30 min. The protein concentration was determined using a BCA assay kit. Protein precipitation was performed using the chloroform–methanol method, which is more effective for removing SDS than other methods, by sequentially adding 1.6 mL methanol, 0.4 mL chloroform, and 1.2 mL water. After centrifugation at 8000 × *g* for 10 min at 4 °C, the precipitated proteins were washed with cold methanol (containing 0.1 M NH_4_OAc) and 80% (v/v) acetone (containing 10 mM NaCl), then air-dried for 5 min. Pellets were resuspended in 500 μL 50 mM ammonium bicarbonate buffer, supplemented with trypsin at a 1:50 (w/w) trypsin-to-protein ratio, and incubated at 37 °C for 12 h. The reaction was acidified with FA to 1% (v/v) final concentration and desalted using peptide desalting spin columns. Peptide concentration was determined using a Pierce Quantitative Colorimetric Peptide Assay, and the samples were vacuum-dried. The resulting powder was stored at − 20 °C.

### High-pH RP-HPLC fractionation (optional step for in-depth identification)

Peptide fractionation was performed using high-pH reversed-phase high-pressure liquid chromatography (high-pH RP-HPLC) to enable in-depth identification. A total of 3.6 mg of digested peptides was fractionated on a Waters XBridge BEH130 C18 column (3.5 μm, 4.6 × 250 mm) on an Ultimate 3000 HPLC system (Dionex, CA, USA) at a flow rate of 1 mL/min. The separation employed buffer A (10 mM ammonium formate, pH 10.0) and buffer B (10 mM ammonium formate with 90% [v/v] ACN, pH 10.0), following a 60-min gradient program (all percentages in v/v): 1% to 25% buffer B over 48 min, increasing to 60% over 5 min, to 70% over 2 min, holding at 70% for 5 min, and returning to 1%. UV absorbance was monitored at 214 nm and 280 nm. Fractions were collected every minute over 60 min, after which these fractions were combined at equal intervals (every 12 min), yielding 12 combined groups, which were then vacuum-dried.

### Enrichment, and LC–MS/MS assay

For both conventional single-shot analysis and in-depth analysis based on fractions obtained by high-pH RP-HPLC above, the enrichment and LC–MS/MS acquisition of PAT-labeled and phosphorylated peptides were performed following an automated online phosphoproteomic method with minor modifications (Zhang et al. 2022). Briefly, the peptide mixture was reconstituted in loading buffer (80% [v/v] ACN, 3% [w/v] trifluoroacetic acid) and loaded onto a phos-trap column for online enrichment. After washing the column with loading buffer to remove non-specifically bound peptides, the target peptides were eluted with 1 M NH_4_H_2_PO_4_ and subsequently trapped on a downstream C18 trap column. The enriched peptides were then resolved on a C18 analytical column (75 μm I.D. × 250 mm, 1.9 μm) (Omicsolution Co., Ltd) using an ACN gradient (5–25%, 0–90 min; 25–35%, 90–105 min; 35–85%, 105–120 min) at a flow rate of 300 nL/min. The source voltage was set to 2.0 kV, and the temperature of the ion transfer tube was maintained at 320 °C. The mass spectrometer operated in data-dependent acquisition (DDA) modes, with MS1 and MS2 being acquired by the Orbitrap detector. MS1 was acquired over an *m/z* range of 400–1300, with 120 K resolution, 60% radio frequency lens, 8 × 10^5^ automatic gain control (AGC) target, and 100 ms maximum ion injection time, and MS2 scans employed a 5 × 10^4^ intensity threshold, 2–6 charge state, 25 s exclusion duration, 54 ms maximum ion injection time, 1.6 Da isolation window, 30% higher-energy collision dissociation energy, automatic scan range, 30 K resolution, 1 × 10^5^ AGC target, and a 3-s duty cycle time. The background ion (C_2_H_6_SiO)_7_H^+^ with *m/z* = 519.1388 was used for internal calibration (lock mass).

### Database search and bioinformatics

Peptide identification and label-free quantification (LFQ) were performed using Proteome Discoverer™ 3.1 software with the SEQUEST search engine. For identification, the “basic” processing workflow (with Percolator node invoked for PSM validation) and the “Basic_ModificationAnalysis” consensus workflow in “Common Templates” were invoked. Notably, in-depth identification required data from each fraction to be imported using “Add Fractions.” For LFQ of technical or biological replicates, data from each sample were imported using “Add Files,” and the “Precursor_Quan_and_LFQ_MPS” processing workflow and the “LFQ_and_Precursor” consensus workflow in “Common Templates” were invoked. Raw files were searched against the BSA FASTA file (P02769 in Swiss-Prot) or the Arabidopsis reference proteome (TAIR10, Ensembl Plants). Trypsin was selected as the enzyme, with a maximum of two missed cleavage sites. Precursor mass tolerance was set at 10 ppm, and fragment mass tolerance was set at 0.02 Da. Variable modifications included 6C-CyaPAT(C), phosphorylation (S, T, Y), oxidization (M), and carbamidomethylation (C), allowing up to five modifications per peptide. False discovery rates (FDR) for peptides were calculated using the Percolator node algorithm, with thresholds of 0.01 for high-confidence and 0.05 for medium-confidence. Phosphorylation and *S*-nitrosylation probability for each site was assessed using the “ptmRS” node with a probability cutoff of 0.75, and sites meeting or exceeding this cutoff were defined as Class I sites. The “Modification analysis” node was used for the presentation of modification sites.

Predicted subcellular localization and functional classification of modified proteins were based on their Gene Ontology (GO) and Kyoto Encyclopedia of Genes and Genomes (KEGG) annotations from omicshare (Gene Denovo Biotechnology Co. Ltd.) and ShinyGO 0.81 (Ge et al. 2019). Protein–protein interaction (PPI) networks were constructed and analyzed with the String database, using clustering analysis (MCL, inflation parameter 3), and visualized in Cytoscape 3.10.

## Supplementary Information

Below is the link to the electronic supplementary material.

Supplementary file1 (XLSX 23 KB)

Supplementary file2 (XLSX 10779 KB)

Supplementary file3 (XLSX 5048 KB)

Supplementary file4 (XLSX 309 KB)

Supplementary file5 (XLSX 276 KB)

Supplementary file6 (XLSX 17 KB)

Supplementary file7 (DOCX 1905 KB)

## Data Availability

The raw data for the comprehensive identification of phosphorylation and *S*-nitrosylation sites in Arabidopsis are available at the Integrated proteome resources repository iProX (http://www.iprox.cn) under project accession number IPX0011065000.
