# Supplementary material for: A novel proteomics workflow for simultaneous analysis of protein phosphorylation and S-nitrosylation
Source: aBIOTECH. 2025 Jul 15;6(3):452–65. doi: 10.1007/s42994-025-00227-2 (PMC12454217; doi:10.1007/s42994-025-00227-2)
Supplement: Supplementary file 7 — Supplementary file7 (DOCX 1905 KB) [file 42994_2025_227_MOESM7_ESM.docx]

**A novel proteomics workflow for simultaneous analysis of protein phosphorylation and *S*-nitrosylation**

Wenyang Zhang^1#^, Yanjiao Wang^1,2#^, Wenyan Li^1^, Shaowen Wu^1^, Yuanyuan Chen^1^, Mingyang Ye^1,2^, Wenjie Huang^1^, Alisdair R. Fernie^3^, Shijuan Yan^1*^

^1^ State Key Laboratory of Swine and Poultry Breeding Industry; Guangdong Key Laboratory for Crop Germplasm Resources Preservation and Utilization, Agro-biological Gene Research Center, Guangdong Academy of Agricultural Sciences, Guangzhou 510640, China

^2^State Key Laboratory for Conservation and Utilization of Subtropical Agro-Bioresources, Guangdong Laboratory for Lingnan Modern Agriculture, College of Life Sciences, South China Agricultural University, Guangzhou 510642 China

^3^Max Planck Institute of Molecular Plant Physiology, Am Muhlenberg 1, 14476, Potsdam-Golm, Germany

^#^equal contribution.

* To whom correspondence may be addressed. E-mail: yan[shijuan@gdaas.cn](mailto:shijuan@agrogene.ac.cn)

**Table of Contents**

Fig. S1 3

Fig. S2 4

Fig. S3 5

Fig. S4 6

Fig. S5 7


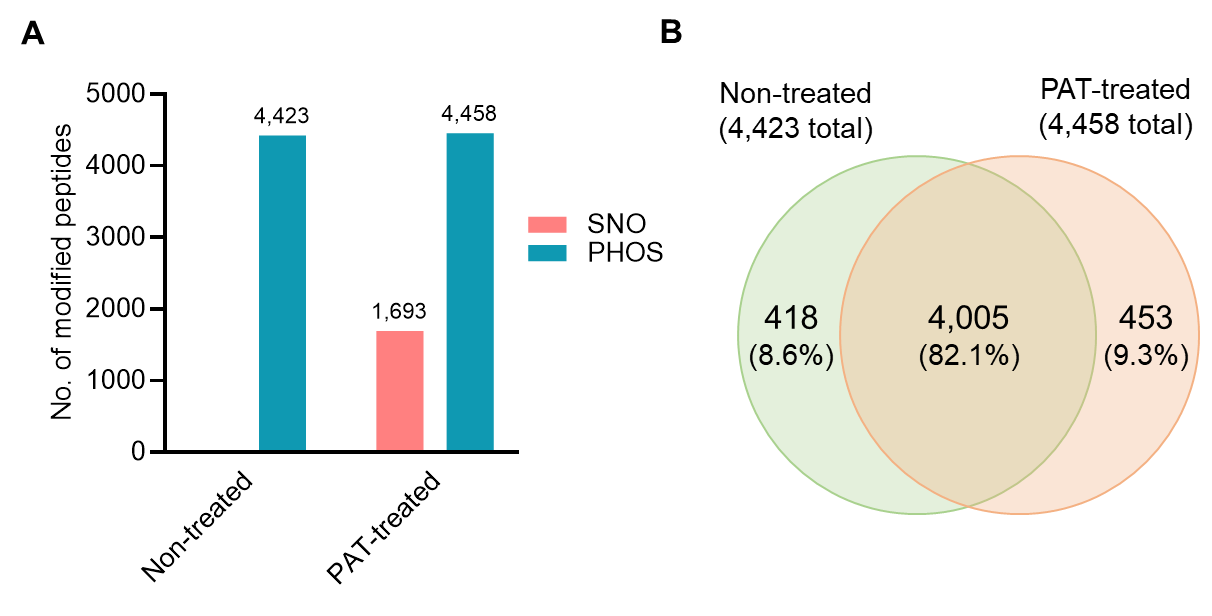


**Fig. S1** **A** The numbers of phosphorylated and *S*-nitrosylated peptides were quantified in technical duplicates for both PAT-treated and untreated samples. **B** The number of phosphopeptides consistently quantified across all samples. All analyses were performed using a lower sample input (150 μg of protein extracted from 30 mg of *Arabidopsis* seedlings)


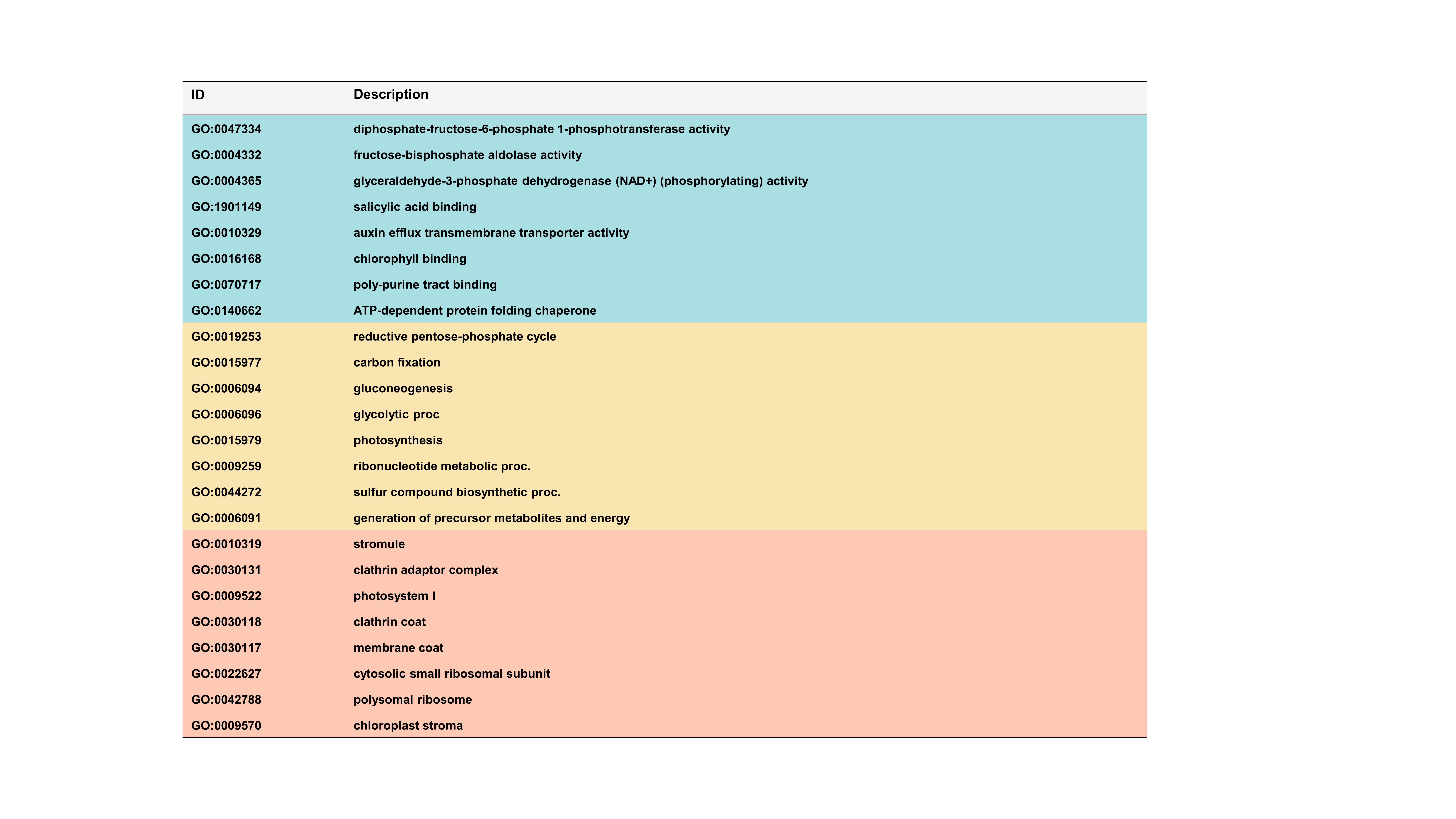


**Fig. S2** The Gene ontology (GO) term IDs and their corresponding detailed names.

**Fig. S3** Optimization of reagent concentration and reaction time for CysPAT labeled. The ordinate represents the number of identified modified peptides

**Fig. S4** The blocking efficiency of free sulfhydryl groups by IAA and CAA

**Fig. S5** The FDR(‱) of search engine on three non-PAT samples
